# Fibroblast growth factor 2 orchestrates angiogenic networking in non-GIST STS patients

**DOI:** 10.1186/1479-5876-9-104

**Published:** 2011-07-06

**Authors:** Thomas K Kilvaer, Andrej Valkov, Sveinung W Sorbye, Eivind Smeland, Roy M Bremnes, Lill-Tove Busund, Tom Donnem

**Affiliations:** 1 Institute of Medical Biology, University of Tromso, PB 9037, Tromso, Norway; 2 Department of Clinical Pathology, University Hospital of North Norway, PB 9038, Tromso, Norway; 3 Institute of Clinical Medicine, University of Tromso, PB 9037, Tromso, Norway; 4 Department of Oncology, University Hospital of North Norway, PB 9038, Tromso, Norway

## Abstract

**Background:**

Non-gastrointestinal stromal tumor soft-tissue sarcomas (non-GIST STSs) constitute a heterogeneous group of tumors with poor prognosis. Fibroblast growth factor 2 (FGF2) and fibroblast growth factor receptor-1 (FGFR-1), in close interplay with platelet-derived growth factor-B (PDGF-B) and vascular endothelial growth factor receptor-3 (VEGFR-3), are strongly involved in angiogenesis. This study investigates the prognostic impact of FGF2 and FGFR-1 and explores the impact of their co-expression with PDGF-B and VEGFR-3 in widely resected tumors from non-GIST STS patients.

**Methods:**

Tumor samples from 108 non-GIST STS patients were obtained and tissue microarrays were constructed for each specimen. Immunohistochemistry was used to evaluate the expressions of FGF-2, FGFR-1, PDGF-B and VEGFR-3.

**Results:**

In the multivariate analysis, high expression of FGF2 (P = 0.024, HR = 2.2, 95% CI 1.1-4.4) and the co-expressions of FGF2 & PDGF-B (overall; P = 0.007, intermediate; P = 0.013, HR = 3.6, 95% CI = 1.3-9.7, high; P = 0.002, HR = 6.0, 95% CI = 2.0-18.1) and FGF2 & VEGFR-3 (overall; P = 0.050, intermediate; P = 0.058, HR = 2.0, 95% CI = 0.98-4.1, high; P = 0.028, HR = 2.6, 95% CI = 1.1-6.0) were significant independent prognostic indicators of poor disease-specific survival.

**Conclusion:**

FGF2, alone or in co-expression with PDGF-B and VEGFR-3, is a significant independent negative prognosticator in widely resected non-GIST STS patients.

## Introduction

Soft-tissue sarcomas (STSs) constitute a group of tumors of mesenchymal lineage, comprising over 50 histological entities [1]. The incidence is low and the lethality is high. With an estimate of 10 000 new cases and nearly 4 000 related deaths in the US in 2010, STSs remain one of the most aggressive types of cancer [2].

Current STS treatment comprises wide resection of the primary tumor with supplementary radiotherapy to those with high-grade lesions [3-5]. Since the use of chemotherapy is a challenge in adult STS, due to controversial efficacy [6], good prognostic and predictive indicators are highly warranted to help select patients for different types of chemotherapy treatments.

Fibroblast growth factors (FGFs) are heparin binding growth factors and as of today there are 18 FGFs and 4 fibroblast growth factor receptors (FGFRs) identified in humans [7]. The most extensive research in this field has been done on FGF2 (also known as basic fibroblast growth factor; bFGF), a growth factor primarily binding FGFR-1 [7]. FGF2 is a known promoter of angiogenesis and lymphangiogenesis [8]. Further, FGF2 stimulates cell growth and migration, but also, in some cases, differentiation [8].

Compared to healthy controls, plasma FGF2 levels, in sarcoma patients, is reported to be elevated. In contrast, low plasma FGF2 levels prior to surgery have been associated with an increased risk of recurrence [9-12]. FGF2 presence has also been confirmed in studies of sarcoma cell-lines [13].

FGF2 has been implicated as a player in different angiogenic and lymphangiogenic pathways [8]. Nissen et al. reported a reciprocal relationship between FGF2 and platelet-derived growth factor-B (PDGF-B) through their corresponding receptors [14]. Kubo et al. found FGF2 induced lymphangiogenesis to be blocked by vascular endothelial growth factor receptor-3 (VEGFR-3) inhibitors [15]. Further, in a study on human umbilical cord endothelial cells grown in the presence of VEGF-A, Welti et al. found FGF2 to rescue angiogenesis in presence of the VEGFR inhibitor Sunitinib^® ^[16].

We have previously reported on the prognostic impact of the PDGFs and VEGFs and their receptors in this cohort of non-GIST STS patients [17,18]. The aim of this study was to investigate the prognostic impact of FGF2 and FGFR-1 expression, and their co-expressions with PDGF-B and VEGFR-3, in widely resected non-GIST STS patients.

## Patients and methods

### Patients and Clinical Samples

Primary tumor tissue from anonymized patients diagnosed with non-GIST STS at the University Hospital of North-Norway and the Hospitals of Arkhangelsk county, Russia, from 1973 through 2006, were collected. In total 496 patients were registered from the hospital databases. Of these, 388 patients were excluded from the study because of: missing clinical data (n = 86), inadequate paraffin-embedded fixed tissue blocks (n = 161) or non-wide resection margins (n = 141). Thus 108 patients with complete medical records, adequate paraffin-embedded tissue blocks and wide resection margins were eligible.

This report includes follow-up data as of September 2009. The median follow-up was 68.4 (range 0.5-391.7) months. Complete demographic and clinical data were collected retrospectively. Formalin-fixed and paraffin-embedded tumor specimens were obtained from the archives of the Departments of Pathology at the University Hospital of North-Norway and the Hospitals of Arkhangelsk County, Russia. The tumors were graded according to the French Fédération Nationale des centres de Lutte Contre le Cancer (FNCLCC) system and histologically subtyped according to the World Health Organization guidelines [1,19]. Wide resection margins were defined as wide local resection with free microscopic margins or amputation of the affected limb or organ.

### Microarray construction

All sarcomas were histologically reviewed by two trained pathologists (S. Sorbye and A. Valkov) and the most representative areas of tumor cells (neoplastic mesenchymal cells) were carefully selected and marked on the hematoxylin and eosin (H/E) slide and sampled for the tissue microarray (TMA) blocks. The TMAs were assembled using a tissue-arraying instrument (Beecher Instruments, Silver Springs, MD). The Detailed methodology has been previously reported [20,21]. Briefly, we used a 0.6 mm diameter stylet, and the study specimens were routinely sampled with four replicate core samples from different areas of neoplastic tissue. Normal tissue from the patients was used as staining control.

To include all core samples, 12 TMA blocks were constructed. Multiple 5-μm sections were cut with a Micron microtome (HM355S) and stained by specific antibodies for immunohistochemistry (IHC) analysis.

### Immunohistochemistry

The applied antibodies were subjected to in-house validation by the manufacturer for IHC analysis on paraffin-embedded material. The antibodies used in the study were FGF2 (rabbit polyclonal; AB1458; Chemicon; 1:200) and FGFR-1 (rabbit polyclonal; sc-121; Santa Cruz; 1:50). The IHC procedures for PDGF-B and VEGFR-3 have been previously described [17,18].

Sections were deparaffinized with xylene and rehydrated with ethanol. Antigen retrieval was performed by placing the specimen in 0.01 M citrate buffer at pH 6.0 and exposed to microwave heating of 10 minutes at 250 W (FGF2) or heated by pressure boiler of 2 minutes (FGFR-1). The DAKO EnVision + System-HRP (DAB) kit was used as endogen peroxidase blocking. As negative staining controls, the primary antibodies were replaced with the primary antibody diluent. Primary antibodies were incubated for 30 minutes (FGF2) or 60 minutes (FGFR-1) in room temperature. The kit DAKO EnVision + System-HRP (DAB) was used to visualize the antigens. This was followed by application of liquid diaminobenzidine and substrate-chromogen, yielding a brown reaction product at the site of the target antigen. Finally, all slides were counter-stained with hematoxylin to visualize the nuclei. For each antibody, included negative staining controls, all TMA staining were performed in a single experiment.

### Scoring of immunohistochemistry

The ARIOL imaging system (Genetix, San Jose, CA) was used to scan the slides of antibody staining of the TMAs. The slides were loaded in the automated slide loader (Applied Imaging SL 50) and the specimens were scanned at low resolution (1.25×) and high resolution (20×) using the Olympus BX 61 microscope with an automated platform (Prior). Representative and viable tissue sections were scored manually on the computer screen semi-quantitatively for cytoplasmic staining. The dominant staining intensity was scored as: 0 = negative; 1 = weak; 2 = intermediate; 3 = strong. All samples were anonymized and independently scored by two trained pathologists (A. Valkov and S. Sorbye). When assessing a variable for a given core, the observers were blinded to the scores of the other variables and to outcome. Mean score for duplicate cores from each individual was calculated separately.

**Figure 1:**
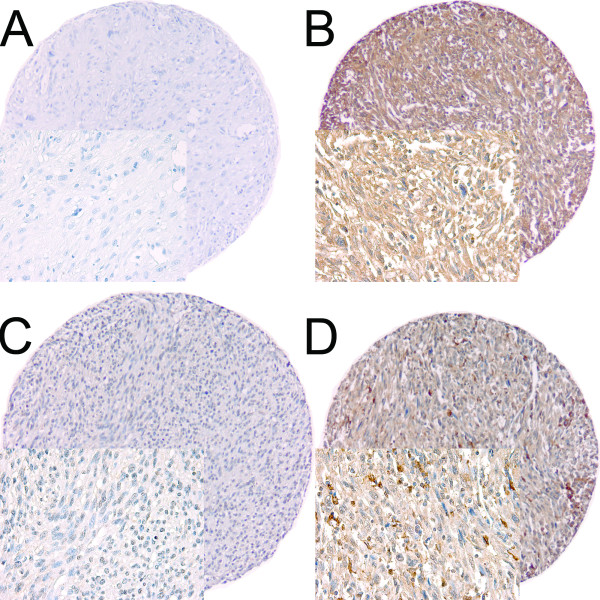
**IHC analysis of TMA of non-gastrointestinal stromal tumor soft-tissue sarcoma representing different scores for tumor cell FGF2 and FGFR-1**. (A) Tumor cell FGF2 low score in Fibrosarcoma; (B) Tumor cell FGF2 high score in undifferentiated pleomorphic sarcoma; (C) Tumor cell FGFR-1 low score in undifferentiated pleomorphic sarcoma; (D) Tumor cell FGFR-1 high score in undifferentiated pleomorphic sarcoma. Abbreviations: FGF, fibroblast growth factor; FGFR, fibroblast growth factor receptor; IHC, immunohistochemistry; TMA, tissue microarray.

High expression in tumor cells was defined as score ≥ 2 (FGF2 and FGFR-1) (Figure 1). The previously published cut-off values for PDGF-B and VEGFR-3 (≥ 1.5) were used to estimate the co-expressions with FGF2 and FGFR-1 [17,18].

### Statistical Methods

All statistical analyses were done using the statistical package SPSS (Chicago, IL), version 16. The IHC scores from each observer were compared for interobserver reliability by use of a two-way random effect model with absolute agreement definition. The intraclass correlation coefficient (reliability coefficient) was obtained from these results. The Chi-square test and Fishers Exact test were used to examine the association between molecular marker expression and various clinicopathological parameters. Univariate analyses were done using the Kaplan-Meier method, and statistical significance between survival curves was assessed by the log-rank test. Disease-specific survival (DSS) was determined from the date of diagnosis to the time of cancer related death. To assess the independent value of different pretreatment variables on survival, in the presence of other variables, multivariate analyses were carried out using the Cox proportional hazards model. Only variables of significant value from the univariate analyses were entered into the Cox regression analysis. Probability for stepwise entry and removal was set at .05 and .10, respectively. The significance level used for all statistical tests was P < 0.05.

### Ethical clearance

The National Data Inspection Board and The Regional Committee for Research Ethics approved the study.

## Results

### Clinopathological Variables

The clinopathological variables are summarized in Table 1. The median age was 57 (range 0-86) years, 56% were female, 73 patients were Norwegian and 35 Russian. The Non-GIST STSs comprised 108 tumors including angiosarcoma (n = 5), fibrosarcoma (n = 8), leiomyosarcoma (n = 34), liposarcoma (n = 13), pleomorphic sarcoma (n = 29), neurofibrosarcoma/malignant peripheral nerve sheath tumor (MPNST, n = 5), rhabdomyosarcoma (n = 6), synovial sarcoma (n = 6) and unspecified sarcoma (n = 2). The tumor origins were distributed as follows: 43% extremities, 19% trunk, 7% retroperitoneal, 4% head/neck and 28% visceral. In addition to surgical resection, 6 patients received both radio-and chemotherapy, 23 patients received chemotherapy alone and 15 patients received radiotherapy alone.

**Table 1:** Prognostic clinicopathological variables as predictors for disease-specific survival in patients with completely resected non-gastrointestinal stromal tumor soft-tissue sarcomas (univariate analyses, log rank test; multivariate analysis, Cox regression analysis)

	Univariate analyses					Multivariate analysis		
**Characteristics**	**Patients** **(n)**	**Patients** **(%)**	**Median** **survival** **(months)**	**5-Year** **survival** **(%)**	**P**	**HR**	**95% CI**	**P**

**Age**								
≤ 20 years	7	7	NR	57	0.960			
21-60 years	55	51	NR	64				
> 60 years	46	43	NR	60				
**Gender**								
Male	47	44	NR	74	0.054			
Female	61	56	127	53				
**Patient nationality**								
Norwegian	73	68	NR	73	< 0.001	1.000		
Russian	35	32	22	38		2.777	1.457-5.292	0.002
**Histological entity**								
Pleomorphic sarcoma	29	27	54	50	0.127			
Leiomyosarcoma	34	32	68	53				
Liposarcoma	13	12	NR	92				
Fibrosarcoma	8	7	NR	88				
Angiosarcoma	5	5	NR	60				
Rhabdomyosarcoma	6	6	NR	67				
MPNST	5	5	NR	80				
Synovial sarcoma	6	6	59	80				
Sarcoma NOS	2	2	NR	100				
**Tumor localization**								
Extremities	46	43	NR	66	0.735			
Trunk	21	19	NR	60				
Retroperitoneum	7	7	NR	71				
Head/Neck	4	4	NR	75				
Visceral	30	28	63	53				
**Tumor size**								
≤ 5 cm	40	37	NR	75	0.262			
5-10 cm	46	43	NR	53				
> 10 cm	20	18	NR	60				
Missing	2	2						
**Malignancy grade**								0.015*
1	32	30	NR	90	< 0.001	1.000		
2	36	33	NR	61		3.808	1.084-13.380	0.037
3	40	37	32	39		5.937	1.721-20.481	0.005
**Tumor depth**								
Superficial	12	11	**	**	0.009	**	**	**
Deep	96	89		57				
**Metastasis at diagnosis**								
No	97	90	218	66	< 0.001	1.000		
Yes	11	10	8	27		4.689	2.004-10.972	< 0.001
**Chemotherapy**								
No	79	73	NR	65	0.234			
Yes	29	27	120	55				
**Radiotherapy**								
No	87	81	NR	63	0.375			
Yes	21	19	NR	57				

Abbreviations: NR, not reached; MPNST, malignant peripheral nerve sheath tumor; NOS, not otherwise specified; *, overall significance as a prognostic factor; **all cases were censored.

### Interobserver variability

Interobserver scoring agreement was tested for FGF2 and FGFR-1. The intraclass correlation coefficients were 0.80 for FGF2 (P < 0.001) and 0.93 for FGFR-1 (P < 0.001), indicating good reproducibility between the investigating pathologists.

### Expression of FGF2/FGFR-1 and their Correlations

FGF2/FGFR-1 expression was localized in the cytoplasm of tumor cells.

FGF2 did not correlate with the clinical variables while low FGFR-1 expression correlated with small tumor size (low expression; < 50 mm 44%, 50-100 mm 34%, > 100 mm 22%, P = 0.005).

### Univariate Analyses

Table 1 summarizes the prognostic impact of the clinopathological variables. Patient nationality (P < 0.001), malignancy grade (P < 0.001), tumor depth (P = 0.009) and metastasis at diagnosis (P < 0.001) were prognostic indicators of DSS.

**Figure 2:**
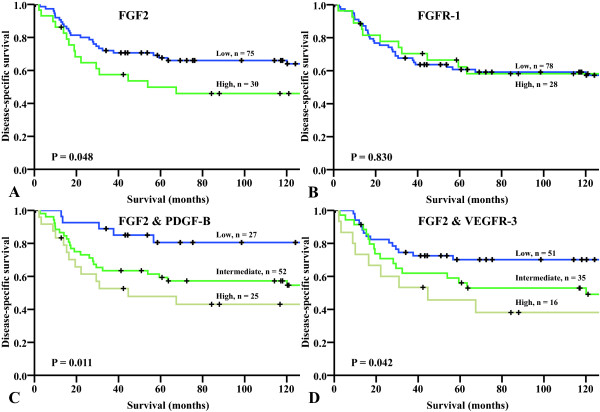
**Disease-specific survival curves for (A) FGF2; (B) FGFR-1; (C) FGF2 & PDGF-B; (D) FGF2 & VEGFR-3**. Abbreviations: FGF, fibroblast growth factor; FGFR, fibroblast growth factor receptor; PDGF, platelet-derived growth factor; VEGFR, vascular endothelial growth factor receptor.

The influence on DSS by FGF2 and FGFR-1 are given in Table 2 and Figure 2 panels A and B. High expression of FGF 2 was significantly (P = 0.048) associated with a poor prognosis.

**Table 2:** Tumor expression of FGF2, FGFR-1 and the co-expressions of FGF2 & PDGF-B and FGF2 & VEGFR-3 and their prediction for disease-specific survival in patients with completely resected non-gastrointestinal stromal tumor soft-tissue sarcomas (univariate analyses, log rank test; multivariate analysis, Cox regression analysis)

	Univariate analyses					Multivariate analysis		
**Marker expression**	**Patients** **(n)**	**Patients** **(%)**	**Median** **survival** **(months)**	**5-Year** **survival** **(%)**	**P**	**HR**	**95% CI**	**P**

**FGF2**								
Low	75	69	NR	68	0.048	1.000		
High	30	28	54	50		2.203	1.108-4.379	0.024
Missing	3	3						
**FGFR-1**								
Low	78	72	NR	61	0.830			
High	28	26	NR	62				
Missing	2	2						
**FGF2 & PDGF-B**								0.007*
Low	27	25	NR	81	0.011	1.000		
Intermediate	52	48	NR	59		3.569	1.311-9.715	0.013
High	25	23	45	48		5.971	1.966-18.132	0.002
Missing	4	4						
**FGF2 & VEGFR-3**								0.050*
Low	51	47	NR	70	0.042	1.000		
Intermediate	35	32	120	56		1.999	0.978-4.087	0.058
High	16	15	45	46		2.584	1.106-6.038	0.028
Missing	6	6						
**FGFR-1 & PDGF-B**								
Low	26	24	NR	75	0.061			
Intermediate	56	52	120	55				
High	23	21	NR	58				
Missing	3	3						
**FGFR-1 & VEGFR-3**								
Low	57	53	NR	66	0.344			
Intermediate	29	27	120	59				
High	18	17	63	52				
Missing	4	4						

Abbreviations: FGF, fibroblast growth factor; FGFR, fibroblast growth factor receptor; NR, not reached; PDGF, platelet-derived growth factor; VEGFR, vascular endothelial growth factor receptor; *overall significance as prognostic factor.

### Multivariate Cox Proportional Hazards Analysis

Table 1 and 2 summarizes the results of the multivariate analysis of clinopathological variables and marker expression, respectively. Russian nationality (P = 0.002), high malignancy grade (P = 0.015), metastasis at diagnosis (P < 0.001) and high FGF2 expression (P = 0.024, HR = 2.203, 95% CI 1.11-4.38) were significant independent negative indicators of DSS.

### Co-expressions

**Figure 3:**
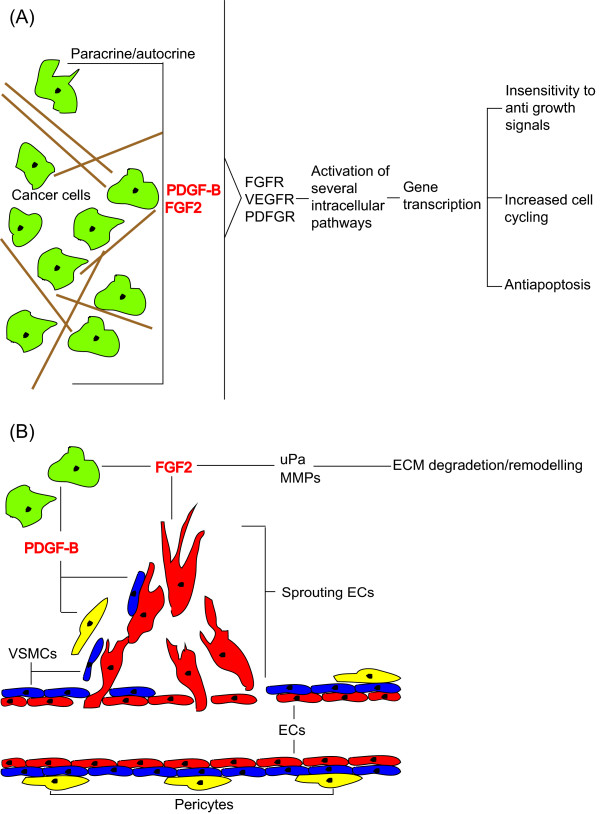
**Proposed mechanisms of stimulation of growth, angiogenesis and motility in non-gastrointestinal stroma tumor soft-tissue sarcomas expressing FGF2, PDGF-B and VEGFR-3**. (A) Paracrin and/or autocrin stimulation of cancer cells leading to activation of intracellular pathways and subsequently survival benefits; (B) FGF2 stimulating angiogenesis through recruitment of endothelial cells and increasing release of MMPs and uPa leading to ECM degradation and remodeling thus enabling tumor cell expansion and motility, PDGF-B recruiting VSMCs and stimulating pericyte coverage of newly formed vessle; Abbreviations: ECM, extracellular matrix; FGF, fibroblast growth factor; FGFR, fibroblast growth factor receptor; PDGF, platelet-derived growth factor; PDGFR, platelet-derived growth factor receptor; MMP, matrix-metallo proteinase; uPa, urokinase-like plasminogen activator; VEGF, vascular endothelial growth factor; VEGFR, vascular endothelial growth factor receptor.

In univariate analyses, the co-expressions of FGF2 & PDGF-B (P = 0.011) and FGF2 & VEGFR-3 (P = 0.042) were significant prognostic indicators of DSS (Table 2). In the multivariate analyses, high expression of FGF2 & PDGF-B was, when compared to low expression, a significant independent prognostic indicator of poor DSS (HR = 6.0, 95% CI = 1.966-18.132, P = 0.002). High expression of FGF2 & VEGFR-3 (HR = 2.6, 95% CI = 1.106-6.038, P = 0.028) was also a significant independent prognosticator (Table 2, Figure 2 panels C and D). Figure 3 shows proposed actions of expressed FGF2, PDGF-B and VEGFRs in non-GIST STSs.

## Discussion

In the study presented herein we have observed high tumor expression of FGF2 and the co-expressions of FGF2 & PDGF-B and FGF2 & VEGFR-3 to be significant, independent and unfavorable prognostic indicators of DSS in non-GIST STS patients with wide resection margins. Few studies have investigated FGF2 and FGFR-1 in STS and no previous studies have reported on the co-expressions with PDGF-B and VEGFR-3. To our knowledge this is the first evaluation of these pathways in non-GIST STSs.

We have previously reported on the prognostic impact of PDGFs and VEGFs in this patient cohort [17,18]. In the previous investigations we found the prognostic impact of these growth factors and their pathways to be dependent on wide resection margins. Without wide resection margins, the prognosis is poor with only 30% 5-year survivors and angiogenic markers could not distinguish between prognostic groups.

Our results are, by large, consistent with previously published data on FGF2 in STSs. Graeven et al. reported that FGF2 levels in serum of STS patients were elevated in comparison to that of controls [12]. Yoon et al., using microarray techniques, found FGF2 gene-expression to be significantly higher in STS patient tissue samples compared to healthy controls [11]. We found high FGF2 expression in tumor to be a significant independent negative prognostic marker in non-GIST STS patients with wide resection margins.

There are several ways in which FGF2 can promote non-GIST STS development, as illustrated in Figure 3. Endothelial cells treated with FGF2 *in vitro *and *in vivo *form tubes, proliferate and are induced to migrate [8]. Further, FGF2 has also been associated with extracellular matrix remodeling, pivotal in angiogenesis/lymphangiogenesis, through increased release and expression of matrix metallo-proteinases and urokinase-like plasminogen activator [8]. In addition, FGF2 has recently been shown to rescue PDGF-B transfected cells undergoing Imatinib^® ^induced apoptosis [22] and to sustain the angiogenic profile of human umbilical cord cells grown with VEGF-A in the presence of the VEGFR inhibitor Sunitinib^® ^[16]. Angiogenesis is one of the hallmarks of cancer and adaptation of an angiogenic profile is one of the deciding steps in carcinogenesis [23]. These latter results indicate that the FGF pathway contributes to the redundancy observed when targeting angiogenesis in cancer (Figure 3b). In addition, FGF2 could function as a growth factor on the tumor cells in a paracrine/autocrine fashion, activating intracellular pathways and ultimately leading cells to proliferate, avoid apoptosis or become insensitive to antigrowth signals (Figure 3a) [8,24].

PDGF-B is an important stabilizer of blood-vessels, working as a chemotactic and proliferative agent on vascular smooth muscle cells (VSMCs) and pericytes [25-27]. Nissen et al. investigated the possibility of interactions between the FGF2 and PDGF-B signaling pathways and found FGF2 and PDGF-B to synergistically induce neovascularization in murine fibrosarcomas [14]. In our cohort, patients who expressed high intensity staining of PDGF-B and FGF2 had HR's of 3.9 or 2.2 [18], respectively, in comparison to those expressing low intensity staining. Examining the co-expression of FGF2 & PDGF-B revealed a HR of 6.0 for the high-high expression group (Table 2), indicating an additive or possibly a synergetic effect of these pathways in non-GIST STSs.

VEGFR-3 is classically associated with lymphangiogenesis, but has recently been linked to angiogenesis [28,29]. Using the mouse corneal assay, Kubo et al. found FGF2 induced angiogenesis to be blocked by administration of VEGFR-3 inhibitors, indicating an interaction between these pathways [15]. Previously, we found non-GIST STS patients with wide resection margins expressing high intensity VEGFR-3 staining to have a HR of 2.0 compared to those with low intensity staining [17]. For the co-expression of FGF2 & VEGFR-3 we found a HR of 2.6 in the high-high expression group, indicating a modest additive effect between these pathways in non-GIST STSs.

FGF2, PDGF-B and VEGFR-3 expression leads to activation of several different intracellular pathways including PI3K, MEKK, SEK, PLCγ and others. Further studies to investigate the relative involvement of these pathways in sarcoma angiogenesis development would be of great interest. Players in these pathways could prove to be targets for novel therapeutic approaches both together with cytokine binding antibodies and receptor blockers and alone.

We have previously found FGF2 and the co-expressions of FGF2 & PDGF-B and FGF2 & VEGFR-3 to be poor independent prognosticators in an unselected large non-small cell lung cancer cohort [30]. The finding of similar results in cancers derived from different embryonic cell-layers may indicate that tumor vasculogenesis as a whole, or at least for certain mechanisms, are universal processes.

## Conclusion

The angiogenic and lymphangiogenic systems have redundant and synergetic properties making it difficult to target these pathways with chemotherapy alone. Nevertheless, we observed that high expression of FGF2 and the co-expressions of FGF2 & PDGF-B and FGF2 & VEGFR-3 are significant independent negative prognostic factors in widely resected non-GIST STS patients. These results suggest that the angiogenic properties of sarcomas are versatile and complex, hence multitargeted antiangiogenic treatment could prove an interesting approach in non-GIST STSs.

## List of abbreviations

bFGF: basic fibroblast growth factor; CI: confidence interval; DSS: disease-specific survival; FGF: fibroblast growth factor; FGFR: fibroblast growth factor receptor; FNCLCC: French Fédération Nationale des centres de Lutte Contre le Cancer; H/E: hematoxylin/eosin; HR: hazard rate; IHC: immunohistochemistry; MMP: matrix metallo proteinase; MPNST: malignant peripheral nerve sheath tumor; Non-GIST STS: non-gastrointestinal stromal tumor soft tissue sarcoma; NOS: not otherwise specified; NR: not reached; PDGF: platelet-derived growth factor; PDGFR: platelet-derived growth factor receptor; STS: soft tissue sarcoma; VEGF: vascular endothelial growth factor; VEGFR: vascular endothelial growth factor receptor; TMA: tissue microarray; uPa: urokinase-like plasminogen activator.

## Competing interests

The authors declare that they have no competing interests.

## Authors' contributions

All authors participated in the study design, result interpretation and in the writing. TK, AV, SS and ES contributed in making the clinical and demographic database. TK, SS and AV scored the cores. TK and TD did the statistical analysis. TK drafted the manuscript. All authors read and approved the final manuscript.

## Funding

This study was funded by the Northern Norway Health Authority, The Norwegian Childhood Cancer Network, The Norwegian Sarcoma Group, The Norwegian Cancer Society and The University of Tromso.

## Aknowlegdements

Thanks to Frode Skjold for coupling of databases, Magnus L. Persson for making the TMA blocks and Helge Stalsberg for helping to collect clinical information.
